# Different regimens for eradication of *Helicobacter pylori* infection in children: a randomized controlled trial

**DOI:** 10.1007/s00431-024-05833-8

**Published:** 2024-11-15

**Authors:** Sana Hosny Barakat, Hind M. Hanafy, Maha Guimei, Elsaid Hamdy Elsawy, Ahmed F. M. Khalil

**Affiliations:** 1 Pediatric Department, Faculty of Medicine, Alexandria University, Alexandria, Egypt; 2 Pathology Department, Faculty of Medicine, University of Alexandria, Alexandria, Egypt

**Keywords:** *Helicobacter pylori*, Ciprofloxacin, Hybrid, Concomitant, Sequential

## Abstract

Eradication of *Helicobacter pylori* (*H. pylori*) infection in children is challenging due to increased antibiotic resistance and decreased effectiveness of the current therapeutic choices, especially in developing countries. The purpose of this study is to compare the efficacy and safety of triple therapy (TT), sequential therapy (ST), hybrid therapy (HT), concomitant therapy (CT), and ciprofloxacin-based triple therapy (CTT) as an empirical therapy for *H. pylori* eradication in children. In this randomized controlled trial, 200 children (aged between 3 and 16 years) with both positive rapid urease test and histopathology for *H. pylori* infection were included. Patients were randomly assigned to receive either TT, ST, HT, CT, or CTT. The eradication status was evaluated using a stool antigen test (SAT) 4 weeks after stoppage of antibiotic therapy and 2 weeks after stoppage of proton pump inhibitors. SAT was performed using an ELISA monoclonal antibody-based kit. The most common presenting symptom was epigastric pain (79%). The most common endoscopic findings were gastric antral erythema (98%) and antral nodularity (54.5%). All gastric biopsies showed superficial lamina propria infiltration with plasma cells and lymphocytes. Active gastritis with neutrophils infiltration was seen in 75% of the cases. Gastric atrophy and intestinal metaplasia were uncommon histopathological findings (8.5% and 1%, respectively). The eradication rates for TT, ST, HT, CT, and CTT were 70%, 77.5%, 80%, 85%, and 90%, respectively, with the latter achieving a statistically significant difference when compared with TT (*p* = 0.025). The rate of occurrence of adverse effects among different regimens was not statistically different.

*Conclusion*: As an empirical treatment for children with *H. pylori* infection, CTT is safe and provides the highest eradication rate. HT, ST, and CT might not be superior to TT.

*Trial registration*: This study was registered at the Pan African Clinical Trials Registry, Cochrane South Africa, under the identifier PACTR202201686010590. Date of registration: 04 January 2022.

**Table:** 

**What is Known:** • Triple therapy has been the standard eradication regimen for pediatric *H. pylori* infection. The efficacy of triple therapy has decreased in many countries due to increased antibiotic resistance.
**What is New:** • This randomized controlled trial is the first to compare triple therapy, sequential therapy, hybrid therapy, concomitant therapy, and ciprofloxacin-based triple therapy for the eradication of pediatric *H. pylori* infection. Triple therapy exhibited the lowest eradication rate among the studied regimens, suggesting it may not be an adequate therapeutic option for infected children. Ciprofloxacin-based triple therapy appears to be a safe and effective therapeutic choice for pediatric *H. pylori* infection. Additionally, this study provides the first reported eradication rate of hybrid therapy in pediatric *H. pylori* infection.

**What is Known:**

• Triple therapy has been the standard eradication regimen for pediatric *H. pylori* infection. The efficacy of triple therapy has decreased in many countries due to increased antibiotic resistance.

**What is New:**

• This randomized controlled trial is the first to compare triple therapy, sequential therapy, hybrid therapy, concomitant therapy, and ciprofloxacin-based triple therapy for the eradication of pediatric *H. pylori* infection. Triple therapy exhibited the lowest eradication rate among the studied regimens, suggesting it may not be an adequate therapeutic option for infected children. Ciprofloxacin-based triple therapy appears to be a safe and effective therapeutic choice for pediatric *H. pylori* infection. Additionally, this study provides the first reported eradication rate of hybrid therapy in pediatric *H. pylori* infection.

## Background

*Helicobacter pylori* (*H. pylori*) infection is one of the most prevalent bacterial infections in children [1]. The triple therapy (TT) has been proposed as a first-line eradication therapy. However, its efficacy has decreased in many countries owing to increased antibiotic resistance [2]. Therefore, other alternative regimens, such as sequential therapy (ST), hybrid therapy (HT), bismuth-based therapy, and concomitant therapy (CT), are now being considered [3]. Recently, researchers also considered the use of less commonly used antibiotics in children, such as fluoroquinolones, because of the global increase in *H. pylori* resistance to metronidazole (MET) and clarithromycin (CLA) [4]. According to a recent worldwide pediatric report, the resistance to MET and CLA has reached up to 91% and 79.6%, respectively [5]. Unfortunately, few pediatric studies on the efficacy of various *H. pylori* therapies have been published in developing countries in general and in Egypt in particular. Moreover, pediatric data evaluating the use of some regimens, like HT and fluoroquinolone-based therapy, are limited. According to a recent pediatric review, the eradication rate of HT had not been reported in children before, while the eradication rate of ciprofloxacin-based triple therapy (CTT) was reported in only one pediatric study [3, 4].

## Aim

The aim of this study was to compare the efficacy and safety of TT, ST, HT, CT, and CTT as an empirical therapy for *H. pylori* eradication in children.

## Material and methods

This randomized controlled trial was conducted from January 2022 to March 2023 at the Pediatric Gastroenterology Clinic and Endoscopy Unit of Alexandria University Children’s Hospital (AUCH), Egypt. Human ethics and consent to participate: children were included in the study only after obtaining formal informed consent from their parents or caregivers. Ethics declaration: the study was carried out in accordance with the institutional review board’s ethical standards and the Helsinki Declaration. The study protocol has been approved by the Ethical Committee of the Faculty of Medicine at Alexandria University in Alexandria, Egypt (number: 0201534). This study was registered at the Pan African Clinical Trials Registry, Cochrane South Africa, under the identifier PACTR202201686010590. The sample size was calculated considering Zhou Y et al.’s pediatric study that showed a first-line *H. pylori* eradication rate of 74.1%, 69.5%, and 84.6% for triple, sequential, and concomitant therapies, similar to the regimens in our study (power 80% and *α* = 0.05) [6]. The sample size was 40 per group. Sample size per group did not need to be increased to control for attrition bias. The study enrolled children who were indicated for upper gastrointestinal endoscopy because they had hematemesis or other persistent upper gastrointestinal symptoms like epigastric pain and vomiting. Exclusion criteria were previous treatment with anti-secretory, antimicrobial, or anti-inflammatory medications within 4 weeks before endoscopy, the presence of a drug allergy to any of the given medications, and non-compliance with drug therapy. The diagnosis of *H. pylori* infection was defined by both a positive rapid urease test (RUT) and by histopathological detection of the organism in gastric biopsies [7]. At least six gastric biopsies were obtained during endoscopy: three from the antrum and three from the corpus. One of the antral biopsies was subjected to a rapid urease test. The other biopsies were sent for histopathological examination. Two sets of 5-μm-thick sections were prepared for each patient; one set was stained by H&E, and the other was Giemsa-stained. *H. pylori* organisms were highlighted using Giemsa stain and, in some cases, using immunohistochemistry. The histopathological changes were described, guided by the updated Sydney classification [8]. Pangastritis was identified when a similar grade of inflammation in the antrum and in the corpus was identified.

Two hundred cases with both positive RUT and positive histology for *H. pylori* were included in the final analysis. The patients were randomly allocated to one of five groups: Group I (*n* = 40): received TT in the form of amoxicillin (AMO), MET, and a proton pump inhibitor (PPI) esomeprazole for 14 days. Group II (*n* = 40): received ST in the form of esomeprazole with AMO for 7 days, followed by esomeprazole with CLA and MET for another 7 days. Group III (*n* = 40): received HT in the form of esomeprazole with AMO for 7 days, followed by esomeprazole with AMO, CLA, and MET for another 7 days. Group IV (*n* = 40): received CT in the form of AMO, esomeprazole, CLA, and MET for 14 days. Group V (*n* = 40): received CTT in the form of ciprofloxacin, esomeprazole, and AMO for 14 days. Monotherapy with PPI (esomeprazole) was continued for 2 weeks after the end of eradication therapy in all studied groups. The drugs used in the treatment protocol included esomeprazole (2 mg/kg/day, maximum 80 mg/day), CLA (15 mg/kg/day, maximum 1 g/day), high-dose AMO (75 mg/kg/day, maximum 3 g/day), MET (20 mg/kg/day, maximum 1 g/day), and ciprofloxacin (20 mg/kg/day, maximum 1 g/day). PPI (esomeprazole) was given at least 30 min before meals. All drugs were divided into two daily doses and given orally. The efficacy of eradication was evaluated at least 4 weeks after the stoppage of antibiotic therapy and at least 2 weeks after the stoppage of the PPI. A negative stool antigen test (SAT) indicated successful eradication [7]. SAT was performed using an ELISA monoclonal antibody-based kit (FORESIGHT® *H. pylori* antigen EIA test kit, Acon laboratories Inc., San Diego, USA).

### Statistical analysis

Data were analyzed using IBM SPSS software package version 20.0. (Armonk, NY: IBM Corp). Categorical data were represented as numbers and percentages. Chi-square test was applied to compare between different groups. Continuous data were tested for normality by Shapiro–Wilk test. Quantitative data were expressed as mean and standard deviation for not normally distributed quantitative variables. Significance was judged at the 5% level.

## Results

**Fig. 1:**
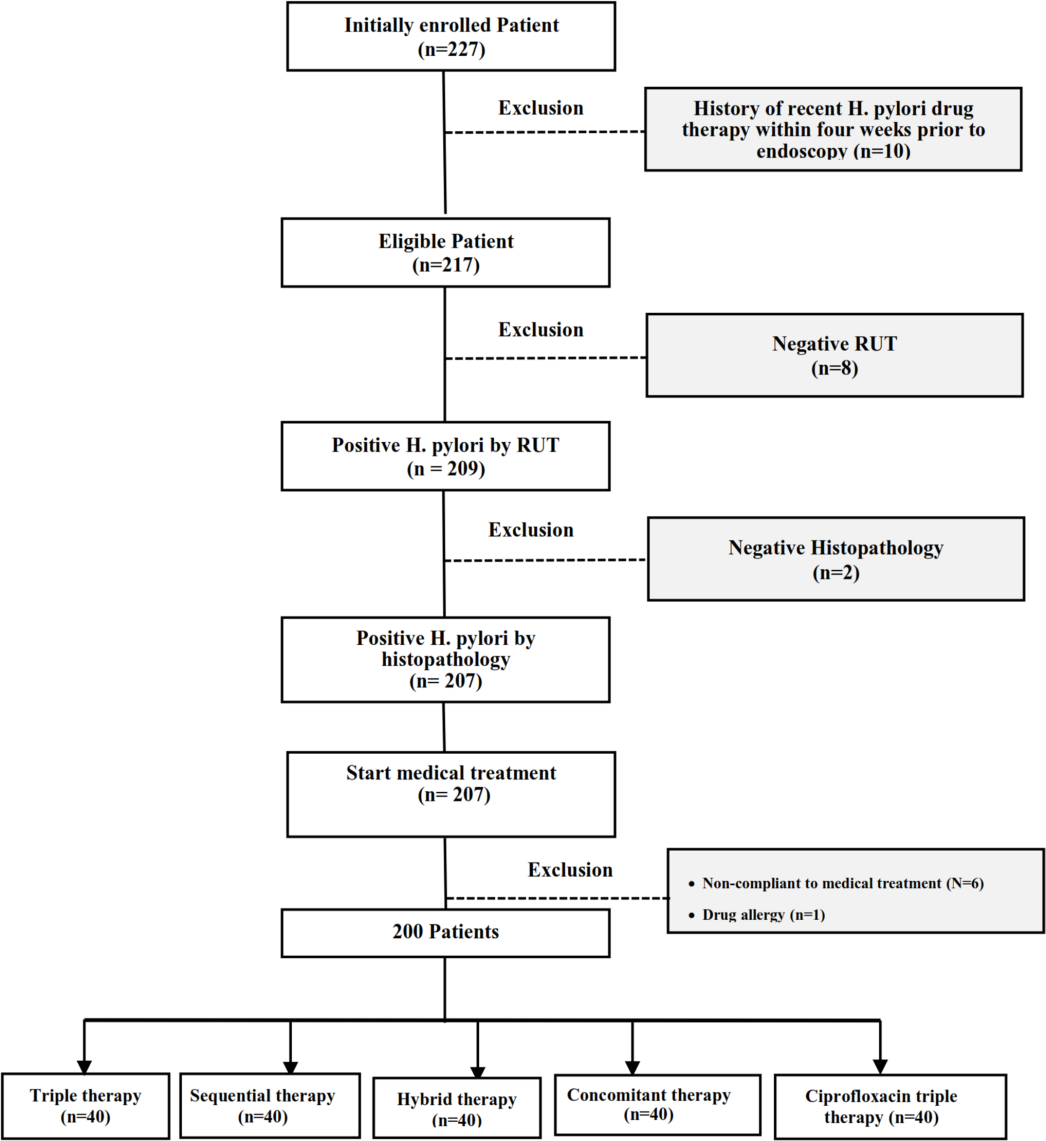
Flowchart demonstrating the cases included in the study

A total of two hundred children were included in the final analysis (102 males and 98 females) out of 227 patients who had an upper endoscopy. The flow diagram showing patient enrollment is shown in Fig. 1.

The age of the patients ranged between 3 and 16 years, with a mean of 7.69 years. Epigastric pain, vomiting, and hematemesis were the chief causes of referral to endoscopy and were reported in 79%, 60.5%, and 35.5% of cases, respectively. On endoscopy, nearly all patients had endoscopic gastric antral erythema (98%), whereas antral nodularity was present in 54.5% of the cases. Peptic ulcers were unusual endoscopic findings (2%). The clinical and endoscopic findings are shown in Table 1.

**Table 1:** The clinical, endoscopic findings, eradication rate, and treatment side effects among the different studied groups

	Triple therapy (*n* = 40)	Sequential therapy (*n* = 40)	Hybrid therapy (*n* = 40)	Concomitant therapy (*n* = 40)	Ciprofloxacin-based triple therapy (*n* = 40)	*p*
Age (years)						
Mean ± SD	6.80 ± 3.55	7.82 ± 4.07	7.53 ± 3.39	7.94 ± 3.68	8.33 ± 3.45	0.339
Sex						
Male	20 (50.0%)	23 (57.5%)	18 (45.0%)	22 (55.0%)	19 (47.5%)	0.787
Female	20 (50.0%)	17 (42.5%)	22 (55.0%)	18 (45.0%)	21 (52.5%)	
Clinical presentation						
Abdominal pain	30 (75.0%)	31 (77.5%)	29 (72.5%)	35 (87.5%)	33 (82.5%)	0.478
Vomiting	23 (57.5%)	28 (70.0%)	26 (65.0%)	20 (50.0%)	24 (60.0%)	0.427
Hematemesis	11 (27.5%)	12 (30.0%)	20 (50.0%)	13 (32.5%)	15 (37.5%)	0.236
Endoscopic findings						
Gastric antral erythema	39 (97.5%)	40 (100%)	39 (97.5%)	40 (100%)	40 (100%)	^MC^p = 1.000
Antral nodularity	20 (50.0%)	18 (45%)	23(57.5%)	26 (65%)	22 (55%)	0.447
Gastric ulcer	2 (5.0%)	1 (2.5%)	0 (0%)	0 (0%)	1 (2.5%)	^MC^p = 0.805
Eradication rate	28 (70.0%)	31 (77.5%)	32 (80.0%)	34 (85.0%)	36 (90.0%)	
p_0_		0.446	0.302	0.108	0.025^*^	
Treatment adverse effects						
Abdominal pain	6 (15.0%)	4 (10.0%)	6 (15.0%)	5 (12.5%)	5 (12.5%)	0.961
Nausea	3 (7.5%)	2 (5.0%)	5 (12.5%)	3 (7.5%)	4 (10.0%)	0.877
Vomiting	5 (12.5%)	4 (10.0%)	7 (17.5%)	6 (15.0%)	6 (15.0%)	0.897
Diarrhea	4 (10.0%)	3 (7.5%)	5 (12.5%)	5 (12.5%)	3 (7.5%)	0.936
Headache	1 (2.5%)	0 (0.0%)	1 (2.5%)	1 (2.5%)	1 (2.5%)	1.000
Metallic taste	7 (17.5%)	4 (10.0%)	4 (10.0%)	9 (22.5%)	2 (5.0%)	0.146

*MC*, Monte Carlo; *SD*, standard deviation

*p*: *p* value for comparing between the different studied groups

*p*_0_: *p* value for comparing between triple therapy and each other group

*Statistically significant at *p* ≤ 0.05

The histopathological evaluation showed antral-predominant gastritis in 68.5% of cases and pangastritis in 31.5% of cases. In antral biopsies, all cases showed superficial lamina propria infiltration with plasma cells and lymphocytes. The grade of inflammation varied between mild, moderate, and severe in 27%, 51.5%, and 21.5% of cases, respectively. Neutrophilic infiltration (indicating activity) was noted in 75% of the cases, also varying in severity from mild (32%), moderate (26%), and severe activity (17%) of all cases. The density of *H. pylori* colonization was mild in 32.5%, moderate in 36.5%, and severe in 31% of the cases. Mild antral mucosal atrophy was detected in 8.5% of the cases. Intestinal metaplasia was detected in only two cases and was also of mild grade. The grading of histopathological findings according to the updated Sydney classification is shown in Table 2.

**Table 2:** The grading of histopathological findings according to updated Sydney classification (*n* = 200)

Histopathological findings in gastric biopsies	No	%
Severity of chronic inflammation		
Mild	54	27.0
Moderate	103	51.5
Severe	43	21.5
Grades of activity		
No activity	50	25
Mild	64	32
Moderate	52	26
Severe	34	17
Mucosal atrophy		
Mild	17	8.5
Moderate	0	0
Severe	0	0
Intestinal metaplasia	2	1.0
*H. pylori* density		
Mild	65	32.5
Moderate	73	36.5
Severe	62	31.0
Predominant site of inflammation		
Antral predominant	137	68.5
Pangastritis (corpus and antrum)	63	31.5

*H. pylori* patients receiving TT showed the lowest rate of eradication (70%), whereas patients in the CTT group showed a 90% eradication rate, followed by the CT group (85%), the HT group (80%), and the ST group (77.5%). The eradication rate in the CTT group was statistically significantly higher than the TT group (*p* = 0.025). Other regimens had a higher eradication rate than the TT, but the results were not statistically significant.

Adverse effects of drug therapy occurred in 22% of cases. Commonly reported side effects were vomiting (14%), abdominal pain (13%), metallic taste (13%), diarrhea (10%), nausea (8.5%), and headache (2%). No joint or other musculoskeletal symptoms were reported in the studied patients. The rate of occurrence of side effects among different regimens was not statistically different. The adverse effects were mild in nearly all cases except for one patient who developed an urticarial skin rash after receiving the TT. This case was excluded from the study. The eradication rate and treatment side effects among the different studied groups are shown in Table 1.

## Discussion

The choice of a proper initial *H. pylori* eradication therapy is usually a difficult task, especially in developing countries where high rates of antibiotic resistance are found [9].

In the present study, CTT achieved the highest eradication rate (90%), and in comparison with TT, it achieved a statistically significantly higher eradication rate (*p* = 0.025). Data about the use of ciprofloxacin in pediatric *H. pylori* infection are limited. In a pediatric Iranian study, Farahmand et al. reported an eradication rate of 87.9% for CTT, which is very similar to our result. The current study and the study by Farahmand et al. are probably the first two studies in the literature to report the eradication rate of a ciprofloxacin-based therapy in children [4]. In adults, the eradication rate of ciprofloxacin-based sequential therapy was 87.6% per protocol analysis compared with 76% in a CLA-based regimen, with no significant differences regarding the adverse effect [10]. The critical issue in prescribing ciprofloxacin to children is always the safety, particularly the risk of arthropathy [11]. In the present study, the rate of occurrence of adverse effects in different regimens was not statistically different. No joint manifestations were reported among any of our studied children. Several studies about the safety of pediatric use of ciprofloxacin revealed a relatively low and reversible risk of arthropathy [12–15]. As ciprofloxacin can be prescribed for resistant infections in children [13], we can apply the treatment regimen described in the current study to patients who are not expected to achieve cure with conventional regimens.

In the current study, the lowest rate of eradication was among the patients in the TT group (70%). Our TT included a PPI, high-dose AMO and MET. Similarly, a pediatric study by Nguyen et al. reported a 69.5% eradication rate for MET-based TT that did not significantly differ from CLA-based TT (72.4%) [16]. In a pediatric Egyptian study, the triple therapy eradication rate was 65% [17]. Studies in adult Egyptians have also confirmed a rate that is nearly similar, ranging between 63.3 and 71.6% at different tertiary centers across the country [18, 19]. Because of these low eradication rates, using the triple therapy as an empirical first line eradication therapy in Egypt should be revised.

ST also had a low eradication rate among our patients (77.5%). Similarly, a pediatric meta-analysis reported that the eradication rates of both TT and ST were lower than expected with both therapies, with an intention to treat 66% and 73%, respectively [20]. Schwarzer et al. showed that the efficacy of ST can vary widely according to CLA and MET susceptibility. They reported an 85.8% eradication rate for ST in cases with no resistance and 28.6% in cases with double resistance [21].

As for the HT, it achieved an eradication rate of 80% in the present study. Scarce data were found about the use of HT in children. A review article published in 2023 about therapeutic choices for pediatric *H. pylori* infection stated that HT has never been used before in children [3]. To the best of our knowledge, this study is the first in the literature to evaluate the use of HT in children. A meta-analysis by Song et al. reported that the eradication rate for HT in adults was 77.6–97.4% in an intention-to-treat (ITT) analysis [22].

CT achieved the second highest eradication rate among our patients (85%). Similarly, in a pediatric comparative study by Zhou et al., CT achieved an eradication rate of 84.5% and was not superior to TT [6]. Consensus reports recommend that CT can be considered as a first-line treatment in regions where susceptibility testing is not available [7]. However, antibiotic resistance can decrease its efficacy. In an adult study, the CT eradication rate was 90% for sensitive strains and only 59% when used with resistant strains [23].

In the current study, the conventional therapeutic regimens (TT, ST, and CT) and also HT did not achieve the recommended goal for *H. pylori* treatment by the consensus guidelines, which is at least 90% [7]. This can be mostly attributed to the increased resistance to CLA and MET. This is evidenced by an Egyptian pediatric study reporting that the *H. pylori* resistance rates to CLA, AMO, and MET were 50%, 20%, and 86.7%, respectively [9]. These higher rates of antibiotic resistance highlight the importance of susceptibility testing for *H. pylori* isolates prior to treatment. Unfortunately, culture-based, tailored therapy is not easily feasible in many developing countries, including Egypt, due to limited resources.

The current study had its own strengths. To the best of our knowledge, this is the first trial of HT for *H. pylori* in the pediatric population. In addition, it is one of two studies in the literature evaluating the eradication rate of CTT for pediatric *H. pylori* and the first study to compare CTT with ST, HT, and CT. However, we experienced some limitations, which included the non-use of culture-based tailored therapy and bismuth’s unavailability. Although we excluded patients who received any prior *H. pylori* eradication therapy, the naïve status of the patients cannot be guaranteed in a developing country with a high *H. pylori* prevalence and frequent antibiotic use. This was another limitation. Despite these limitations, the study expands the field of research for a condition with high morbidity and advocates for a revision of first-line eradication therapy, especially in developing countries with high antibiotic resistance. The study also highlights new empirical therapeutic choices that could be considered when tailored *H. pylori* eradication therapy is not feasible.

## Conclusion

In our study, CTT achieved the highest eradication rate, which was significantly higher than TT. ST, HT, and CT had a relatively higher eradication rate than TT, but the difference was not statistically significant. All regimens were generally well tolerated, with no severe adverse effects or significant differences between the different regimens.

## Data Availability

No datasets were generated or analysed during the current study.
